# Sustained Axonal Degeneration in Prolonged Disorders of Consciousness

**DOI:** 10.3390/brainsci11081068

**Published:** 2021-08-14

**Authors:** Sergio Bagnato, Maria Enza D’Ippolito, Cristina Boccagni, Antonio De Tanti, Lucia Francesca Lucca, Antonio Nardone, Pamela Salucci, Teresa Fiorilla, Valeria Pingue, Serena Gennaro, Maria Ursino, Valentina Colombo, Teresa Barone, Francesca Rubino, Maria Andriolo

**Affiliations:** 1 Unit of Neurophysiology and Unit for Severe Acquired Brain Injuries, Rehabilitation Department, Giuseppe Giglio Foundation, 90015 Cefalù, Italy; cristina.boccagni@hsrgiglio.it (C.B.); terryfiorilla@yahoo.it (T.F.); francesca.rubino@hsrgiglio.it (F.R.); 2 Molecular Biology Laboratory, Giuseppe Giglio Foundation, 90015 Cefalù, Italy; astutapc@yahoo.it (M.E.D.); maria.andriolo@hsrgiglio.it (M.A.); 3 Cardinal Ferrari Center, 43012 Fontanellato, Italy; antonio.detanti@centrocardinalferrari.it (A.D.T.); serena.gennaro@centrocardinalferrari.it (S.G.); 4 RAN (Research in Advanced Neuro-Rehabilitation), S. Anna Institute, 88900 Crotone, Italy; l.lucca@istitutosantanna.it (L.F.L.); m.ursino@istitutosantanna.it (M.U.); 5 Neurorehabilitation and Spinal Units, ICS Maugeri, Institute of Pavia, 27100 Pavia, Italy; antonio.nardone@icsmaugeri.it (A.N.); valeria.pingue@icsmaugeri.it (V.P.); 6 Montecatone Rehabilitation Institute, 40026 Imola, Italy; pamela.salucci@montecatone.com (P.S.); valentina.colombo@montecatone.com (V.C.); 7 Immunohematology and Transfusion Service, 90015 Cefalù, Italy; barone.teresa@asppalermo.org

**Keywords:** neurofilament light chain, traumatic brain injury, hypoxic-ischemic brain injury, unresponsive wakefulness syndrome, vegetative state, minimally conscious state, secondary brain injury, biomarkers of brain injury

## Abstract

(1) Background: Sustained axonal degeneration may play a critical role in prolonged disorder of consciousness (DOCs) pathophysiology. We evaluated levels of neurofilament light chain (NFL), an axonal injury marker, in patients with unresponsive wakefulness syndrome (UWS) and in the minimally conscious state (MCS) after traumatic brain injury (TBI) and hypoxic-ischemic brain injury (HIBI). (2) Methods: This prospective multicenter blinded study involved 70 patients with prolonged DOC and 70 sex-/age-matched healthy controls. Serum NFL levels were evaluated at 1–3 and 6 months post-injury and compared with those of controls. NFL levels were compared by DOC severity (UWS vs. MCS) and etiology (TBI vs. HIBI). (3) Results: Patients’ serum NFL levels were significantly higher than those of controls at 1–3 and 6 months post-injury (medians, 1729 and 426 vs. 90 pg/mL; both *p* < 0.0001). NFL levels were higher in patients with UWS than in those in MCS at 1–3 months post-injury (*p* = 0.008) and in patients with HIBI than in those with TBI at 6 months post-injury (*p* = 0.037). (4) Conclusions: Patients with prolonged DOC present sustained axonal degeneration that is affected differently over time by brain injury severity and etiology.

## 1. Introduction

Severe acute brain injuries, typically traumatic brain injury (TBI) and hypoxic-ischemic brain injury (HIBI), disrupt the ability of the brain to support consciousness, leading to a disorder of consciousness (DOC). Prolonged DOC [1], i.e., unresponsive wakefulness syndrome (UWS; sleep/wake cycles but no sign of awareness) [2] or a minimally conscious state (MCS; minimal and fluctuating signs of awareness), is defined as consciousness impairment lasting for more than 28 days [3]. Acute brain injuries, especially TBI, may trigger persistent neuronal loss leading to secondary brain damage, which further affects the chances of recovery in patients with prolonged DOC [4]. In particular, neuroimaging studies have shown that moderate to severe TBI induces progressive brain-volume loss involving the gray and white matter, although the mechanisms underlying this postinjury atrophy are not well understood [5,6]. Similarly, neuropathological studies have demonstrated broad white-matter damage of traumatic and nontraumatic etiologies that cannot be explained entirely by acute injury in patients with prolonged DOC [7,8].

Biomarkers could be useful in the evaluation of persistent secondary brain damage in patients with prolonged DOC, as they may enable the definition of specific neuroprotective strategies. In this context, neurofilaments are potential candidates. Neurofilaments, composed of light-, medium-, and heavy-molecular-weight proteins, are the dominant intermediate filaments of the neural cytoskeleton and play an important role in the maintenance of axon structure and function [9]. Neurofilament light chain (NFL) levels, which can be evaluated in cerebrospinal fluid and peripheral blood, are increased in several diseases characterized by acute or chronic neuronal damage, such as TBI [10], HIBI after cardiac arrest [11], subarachnoid hemorrhage [12], stroke [13], amyotrophic lateral sclerosis [14], Alzheimer’s disease [15], and in patients with Parkinson’s disease and cognitive impairment [16]. In particular, serum NFL levels are markedly increased in the acute phase of TBI and HIBI, and they correlate with poor outcomes [10,11]. Thus, NFL is a promising biomarker of axonal injury and neurodegeneration, potentially related to the amount of brain injury and disease progression [17,18,19].

In a previous study, we found high NFL levels in the cerebrospinal fluid of patients with post-traumatic DOC [20]; nevertheless, many questions remain unanswered, as we did not investigate non-traumatic etiologies (e.g., HIBI) or differences among patients with different DOCs or collect follow-up data. Moreover, the assessment of NFL in cerebrospinal fluid is not suitable for routine clinical use because of the difficulty of obtaining samples by lumbar puncture. Thus, this multicenter prospective longitudinal study was designed to evaluate: (i) whether serum NFL levels in patients with prolonged DOC differ from those in matched healthy controls at 1–3 months (baseline) and/or 6 months post-injury, (ii) whether these levels differ according to DOC severity (UWS or MCS) and/or etiology (TBI or HIBI), and (iii) whether serum NFL levels are related to 6-month outcomes. Results from this study may improve characterization of the mechanisms of long-term brain injury in patients with prolonged DOC and the usefulness of NFL as a potential biomarker of UWS and MCS severity and outcome. 

## 2. Materials and Methods

### 2.1. Participants 

For this study, patients admitted to five Italian centers specialized in the rehabilitation of patients with prolonged DOC following acute brain injury were recruited between June 2019 and February 2020. Inclusion criteria were: (i) diagnosis of UWS or MCS according to the Coma Recovery Scale—Revised (CRS-R) [21] at the time of study inclusion, (ii) DOC caused by TBI or HIBI, (iii) study inclusion at 28 days–3 months after acute brain injury, and (iv) age of 18–65 years. Exclusion criteria were: (i) previous history of acute brain injury, pathology affecting the myelin (e.g., multiple sclerosis), or neurodegenerative or psychiatric disease; (ii) previous history of cancer; (iii) unstable clinical condition (e.g., hemodynamic instability, severe respiratory failure, or acute hydrocephalus). Patients’ data were compared with those of sex- and age-matched healthy controls with no previous history of neurological, psychiatric, or neoplastic disease who were recruited at the coordinating center among blood donors and healthcare personnel.

### 2.2. Clinical Evaluation

At the time of study inclusion, all patients underwent standard neurological examination and assessment with the CRS-R over three consecutive days, with the CRS-R administered at least five times. The CRS-R is used to diagnose UWS, MCS, and emergence from MCS and is the most reliable tool available for the assessment of consciousness in patients with DOC following coma [22]. It consists of 23 hierarchically organized items grouped into six subscales addressing auditory, visual, motor, oromotor/verbal, communication, and arousal functions. Total CRS-R scores range from 0 (comatose state) to 23 (emergence from MCS). To reduce the risk of misdiagnosis, diagnoses of UWS and MCS were accepted only when confirmed at all five evaluations [23]. The same 3-day evaluation with five CRS-R administrations was repeated 6 months after brain injury. Six-month outcomes were characterized using the Glasgow Outcome Scale—Extended (GOSE) [24], a global 1–8 scale with the categories of death (score of 1), vegetative state, severe disability (lower and upper), moderate disability (lower and upper), and good recovery (lower and upper; scores of 7 and 8).

### 2.3. NFL Analysis

At the end of the initial and follow-up clinical evaluations, blood samples for NFL analysis were collected from the patients by venipuncture into serum-separating tubes, centrifuged at 1500 rpm for 15 min, and stored at −80 °C until use. Blood samples from healthy controls were collected the day of the inclusion in the study. Samples collected outside the coordinating center were sent to the coordinating center, where the serum was aliquoted and analyzed using a commercially available NFL enzyme-linked immunosorbent assay (ELISA) kit as described by the manufacturer (Uman Diagnostics, Umeå, Sweden) for cerebrospinal fluid analysis. With this ELISA assay, serum NFL levels are correlated to those in cerebrospinal fluid and to results obtained with other platforms for serum NFL analysis [25]. The researchers who performed the NFL analysis were blinded to the clinical data, and those who performed the clinical evaluations were blinded to the NFL data.

### 2.4. Sample Size Calculation

The preliminary sample size calculation was performed with consideration of our previous study [20], in which mean NFL levels in cerebrospinal fluid were about 5.7 times higher in 10 patients with prolonged DOC than in a control population of patients with Alzheimer’s disease. We postulated that the magnitude of this difference could be maintained for the serum NFL levels of patients and healthy controls, assuming an alpha error level of 5% and a beta error level of 50%. This postulate was rather stringent, as the patients with Alzheimer’s disease had higher NFL levels than did the healthy subjects recruited for this study. Based on these criteria, the required sample size was 38 participants (19 patients and 19 controls). However, the effective sample size for the study was about 3.7 times larger (70 patients and 70 controls), as we planned to perform comparisons of patient subpopulations. The sample size calculation was performed with G*Power, version 3.1 [26].

### 2.5. Statistical Analysis

Demographic and clinical data are expressed as means (SD); NFL levels are expressed as medians and 25th and 75th percentiles. The Mann–Whitney *U* test (for continuous variables) and Fisher’s exact test (for categorical variables) were used to compare demographic and clinical data between patients and healthy controls and between subgroups of patients (UWS vs. MCS, TBI vs. HIBI). We used Spearman’s rank-correlation test to examine whether NFL levels correlated with GOSE scores at 6 months post-injury. *p* values < 0.05 were considered to be significant. The statistical analysis was performed with Prism (GraphPad Software), version 9.1.1. 

## 3. Results

### 3.1. NFL Levels in Patients and Controls

**Figure 1:**
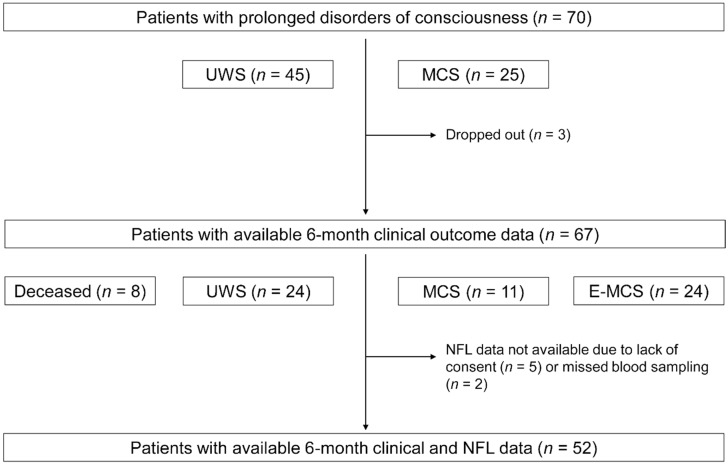
Study flow and patients’ levels of consciousness at 1–3 and 6 months post-injury. UWS, unresponsive wakefulness syndrome; MCS, minimally conscious state; E-MCS, emergence from a minimally conscious state; NFL, neurofilament light chain.

Seventy patients with prolonged DOC and 70 sex- and age-matched healthy controls were recruited (Table 1 and Supplementary Table S1). Forty-five patients had UWS and 25 patients were in MCS. DOCs were caused by TBI in 48 patients (37 road accidents, nine falls, and two assaults) and by HIBI in 22 patients (20 cardiac arrests and two asphyxias). NFL and other clinical data were available for all 70 patients included in the study. Six-month outcomes were available for 67 patients (including eight patients who died before the follow-up evaluation); three patients dropped out of the study because they were discharged and not available for the 6-month follow-up evaluation. The 6-month clinical evaluation was completed for 59 patients, and NFL data were available for 52 of these patients (Figure 1).

**Figure 2:**
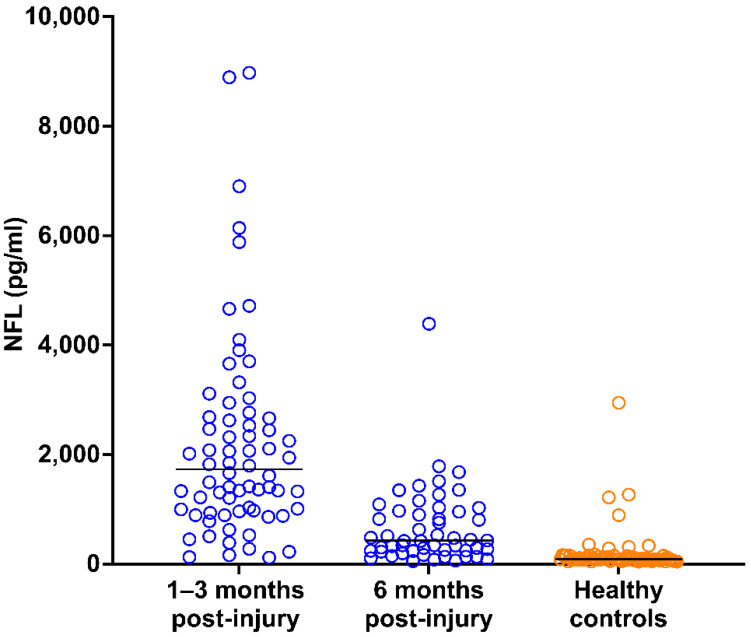
Distribution of NFL values in patients (at 1–3 and 6 months post-injury) and healthy controls. The horizontal lines indicate median values. One datapoint in the 1–3 months group is not reported because it fell outside of the axis limit. NFL, neurofilament light chain.

Patients’ serum NFL levels at 1–3 months (median, 1729 pg/mL; 25th and 75th percentiles, 982 and 2679 pg/mL, respectively) and 6 months (median, 426 pg/mL; 25th and 75th percentiles, 241 and 959 pg/mL, respectively) post-injury were significantly higher than those of healthy controls (median, 90 pg/mL; 25th and 75th percentiles, 74 and 131 pg/mL, respectively; *U* = 159 and 481, respectively; both *p* < 0.0001; Figure 2). Moreover, patients’ serum NFL levels were significantly higher at 1–3 months than at 6 months post-injury (*U* = 586, *p* < 0.0001; Figure 2). Comparisons of NFL levels at 1–3 months and 6 months post-injury in patient subgroups are provided in Table 2.

### 3.2. NFL Levels in Patients with Different States of Consciousness

**Figure 3:**
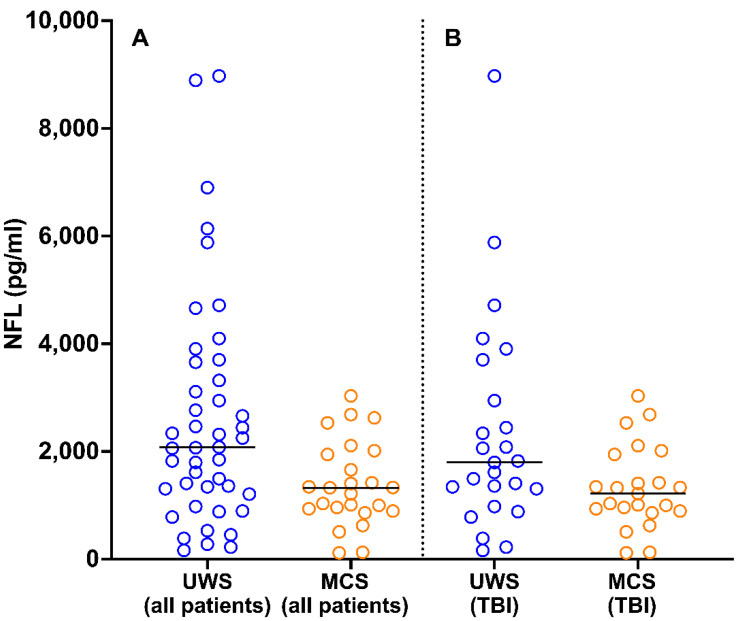
Distribution of NFL values in patients with UWS and MCS in the total sample (**A**) and among patients with TBI (**B**). The horizontal lines indicate median values. One datapoint in the UWS (all patients) group is not reported because it fell outside of the axis limit. NFL, neurofilament light chain; UWS, unresponsive wakefulness syndrome; MCS, minimally conscious state; TBI, traumatic brain injury.

At the time of study inclusion, NFL levels were higher in patients with UWS (median, 2080 pg/mL; 25th and 75th percentiles, 1308 and 3658 pg/mL, respectively) than in those in MCS (median, 1325 pg/mL; 25th and 75th percentiles, 934 and 3269 pg/mL, respectively; *U* = 347, *p* = 0.008; Figure 3A). This finding was confirmed in a more homogeneous subgroup of patients; among patients with TBI (*n* = 48), NFL levels were higher in those with UWS (*n* = 25; median, 1797 pg/mL; 25th and 75th percentiles, 1308 and 2945 pg/mL, respectively) than in those in MCS (*n* = 23; median, 1218 pg/mL; 25th and 75th percentiles, 913 and 1680 pg/mL, respectively; *U* = 189, *p* = 0.04; Figure 3B).

Of the 52 patients for whom 6-month follow-up NFL data were available, 22 had UWS and 30 were conscious (11 in MCS and 19 emerged from MCS). Six-month post-injury NFL levels did not differ between patients with UWS (median, 434 pg/mL; 25th and 75th percentiles, 250 and 997 pg/mL, respectively) and those with MCS and emergence from MCS (median, 426 pg/mL; 25th and 75th percentiles, 238 and 919 pg/mL, respectively; *U* = 314, *p* = 0.8).

### 3.3. NFL Levels in Patients with Prolonged DOC of Different Etiologies

At the time of study inclusion, the NFL levels of patients with HIBI (median, 2545 pg/mL; 25th and 75th percentiles, 1708 and 3574 pg/mL, respectively) were higher than those of patients with TBI (median, 1383 pg/mL; 25th and 75th percentiles, 954 and 2165 pg/mL, respectively; *U* = 331, *p* = 0.01). This result can be explained by the higher proportion of patients with UWS among those with HIBI (*n* = 20/22 (91%)) than among those with TBI [*n* = 25/48 (52%)]. Among patients with UWS, NFL levels did not differ between those with HIBI (median, 2564 pg/mL; 25th and 75th percentiles, 1687 and 3910 pg/mL, respectively) and those with TBI (median, 1797 pg/mL; 25th and 75th percentiles, 1308 and 2945 pg/mL, respectively; *U* = 190, *p* = 0.2).

**Figure 4:**
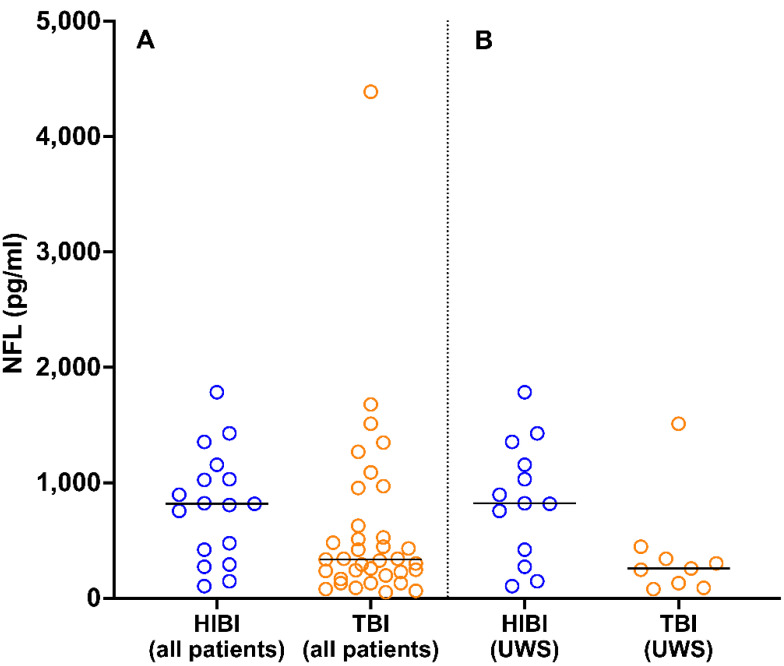
Distribution of NFL values 6 months post-injury in patients with HIBI and TBI in the total sample (**A**) and among patients with UWS (**B**). The horizontal lines indicate median values. NFL, neurofilament light chain; HIBI, hypoxic-ischemic brain injury; TBI, traumatic brain injury; UWS, unresponsive wakefulness syndrome.

Of the 52 patients for whom 6-month follow-up NFL data were available, 35 had TBI and 17 had HIBI. At 6 months post-injury, the NFL levels of patients with HIBI (median, 818 pg/mL; 25th and 75th percentiles, 421 and 1031 pg/mL, respectively) were higher than those of patients with TBI (median, 337 pg/mL; 25th and 75th percentiles, 212 and 577 pg/mL, respectively; *U* = 191, *p* = 0.04; Figure 4A). This finding was not affected by the difference in the proportions of patients with UWS in the HIBI (*n* = 13/15 (87%)) and TBI (*n* = 9/18 (50%)) groups; among patients with UWS, NFL levels were higher in those with HIBI (median, 823 pg/mL; 25th and 75th percentiles, 421 and 1157 pg/mL, respectively) than in those with TBI (median, 257 pg/mL; 25th and 75th percentiles, 131 and 342 pg/mL, respectively; *U* = 27, *p* = 0.04; Figure 4B).

### 3.4. Six-Month Outcomes and Correlation with NFL Levels

Among the 67 patients for whom 6-month clinical outcomes were available, GOSE scores were 1 in eight patients, 2 in 31 patients, 3 in 17 patients, 4 in two patients, 5 in two patients, 6 in four patients, and 7 in three patients. Six months post-injury, CRS-R and GOSE scores were higher for patients in MCS than for those with UWS at admission, and for patients with TBI than for those with HIBI (Table 1). Baseline NFL levels did not correlate with 6-month GOSE scores in the total sample (*r* = −0.004, *p* = 0.97) or in subgroups of patients with UWS, MCS, TBI, and HIBI (all *p* > 0.05).

## 4. Discussion

In this study, we found that the serum NFL levels of patients with prolonged DOC after TBI or HIBI were more than 19 times higher than those of matched healthy controls at 1–3 months post-injury, and almost 5 times higher at 6 months post-injury. This increase was related to the severity and etiology of brain injury, in different ways depending on the time elapsed since injury; serum NFL levels were higher in patients with UWS than in those in MCS at 1–3 months post-injury, and in patients with HIBI than in those with TBI at 6 months post-injury. Finally, serum NFL levels at 1–3 months post-injury did not correlate with 6-month outcomes.

The pronounced serum NFL elevation seen in patients with prolonged DOC suggests that severe brain injuries can trigger neurodegeneration with axonal damage lasting at least 6 months post-injury. The mechanisms responsible for the massive release of NFL release into the blood at 1–3 months after severe TBI or HIBI are poorly understood, but some hypotheses can be offered. Axonal injury itself can trigger neurodegeneration and white matter atrophy [27], likely leading to progressive proteinopathies associated with neuronal toxicity and death [28]. In patients with prolonged DOC, the cerebrospinal fluid level of amyloid-β is reduced [29], which may result in predisposition to amyloid-β plaque formation and then neuronal loss leading to NFL release. Axonal injury with NFL release also can be induced by the sustained neuroinflammation triggered by acute brain injury. Long-term microglial activation has been reported after moderate to severe TBI and related to neurodegeneration with white matter atrophy [30,31]. Moreover, animal models and human autoptic data suggest that TBI causes blood–brain barrier dysfunction that persists for several years [32] and may increase NFL release into the blood [33,34]. We also found remarkable evolution of the serum NFL level 6 months post-injury, which may reflect progressive mitigation of the processes involved in neurodegeneration and neuronal death in patients with prolonged DOC. An alternative explanation is that the reduction in the serum NFL level at 6 months post-injury is caused by progressive brain atrophy with the reduction in the number of axons susceptible to further degeneration with NFL release. The latter hypothesis is supported by autoptic [7] and neuroimaging [35] findings of marked white matter atrophy in patients with prolonged DOC and by a biomarker study showing reduced baseline release of neuron-specific enolase at 1 year after severe TBI, possibly because of brain atrophy [36].

Among all patients and those with TBI, patients with the most severe DOC (UWS) had higher serum NFL levels at 1–3 months post-injury than patients in MCS. Compared with patients in MCS, those with UWS have more severe impairment of the complex networks connecting the cortical areas, basal ganglia, and thalamus that support consciousness [37,38,39]. As NFL is expressed mainly in large myelinated axons [40], these higher NFL levels in patients with UWS likely indicate more marked degeneration of the projection neurons involved in consciousness. Moreover, this finding supports the idea of a specific time window in which marked axonal degeneration occurs in patients with UWS relative to those in MCS, which further compromises the chance of recovery from this most severe consciousness impairment. Thus, future neuroprotective strategies aiming to reducing axonal loss and neuronal death should be implemented in the early phase of prolonged DOC, especially in patients with UWS.

Patients with prolonged DOC etiologies underlain by different pathophysiological mechanisms had similar NFL levels in the early phase of prolonged DOC; at 6 months post-injury, patients with HIBI had higher NFL levels than did those with TBI, reflecting a slower decrease in NFL levels over time. This finding was unexpected; we expected to find that more NFL release had occurred in patients with TBI at both timepoints, as result of slow Wallerian degeneration triggered by the post-traumatic diffuse axonal injury. Recent studies have revealed high serum NFL levels in patients with HIBI immediately after cardiac arrest, especially in cases of poor outcomes, but no data for post-acute phases or comparison of patients with DOC of different etiologies have been reported [11,41]. The processes involved in long-term NFL release after HIBI are not well understood, but data from neonatal patients with HIBI suggest that chronic inflammation, gliosis, and epigenetic dysregulation cause sustained brain injury lasting weeks, months, and even years after hypoxic insult [42,43].

We found that patients in MCS at 1–3 months post-injury had better 6-month outcomes than did patients with UWS, and that patients with TBI had better outcomes than did patients with HIBI. These findings are in agreement with current knowledge about the prognosis of patients with prolonged DOC [44]. Although serum NFL levels at the time of study inclusion were higher in patients with the most severe DOC, they did not correlate with GOSE-classified outcomes. A possible explanation for this finding is that the GOSE allows for very general outcome evaluation, preventing the assessment of correlations between NFL levels and performance in specific cognitive domains. Accordingly, further studies with more accurate assessment of the prognostic significance of serum NFL levels in patients with prolonged DOC, using tools enabling detailed functional and neuropsychological evaluation, are mandatory.

This study has some limitations. The conventional ELISA kit that we used is less sensitive than the single-molecule array method for serum NFL detection [25]; however, the single-molecule array instrument still has limited availability in routine-practice laboratories. Comparisons between subpopulations were performed in small numbers of patients. Moreover, the temporal window of 6 months adopted for clinical and NFL evaluations was adequate for the evaluation of final outcomes in most patients with HIBI, but some patients with TBI may show consciousness improvement up to 1 year after brain injury [44].

## 5. Conclusions

Patients with prolonged DOC are characterized by sustained NFL release that is differently affected at distinct timepoints by the severity and etiology of brain injury. This study supports the occurrence of long-term neurodegeneration that counteracts the plastic reorganization required for the recovery of consciousness and functional independence and, thus, may have detrimental effects on outcomes in patients with UWS and MCS. Secondary neurodegeneration after severe brain injury cannot be considered an ineluctable destiny, and serum NFL can be used as a biomarker to track axonal degeneration in clinical trials evaluating new neuroprotective treatments for patients with prolonged DOC.

## Figures and Tables

**Table 1:** Participant characteristics.

Characteristic	All Patients (*n* = 70)	Healthy Controls (*n* = 70)	*p*	UWS (*n* = 45)	MCS (*n* = 25)	*p*	TBI (*n* = 48)	HIBI (*n* = 22)	*p*
Males	58	58	1	38	20	0.7	41	17	0.5
Females	12	12		7	5		7	5	
Age (years)	39.2 (14.8)	39.4 (14.5)	0.9	41.6 (14.6)	35.3 (14.8)	0.08	37 (14.7)	44 (14.2)	0.06
Time since brain injury (days)	47.2 (20)			45.4 (20)	50.8 (20.3)	0.2	47.5 (19.4)	46.7 (21.9)	0.8
CRS-R score									
1–3 months post-injury	7.2 (4.7)			4.3 (1.6)	12.4 (3.9)	**<0.0001**	8.2 (5.2)	4.8 (1.8)	**0.02**
6 months post-injury	13.3 (8.2)			9 (6.6)	19.8 (5.9)	**<0.0001**	15.9 (8)	6.9 (4.5)	**<0.0001**
GOSE score	2.4 (1.5)			2.4 (1.3)	3.3 (1.7)	**0.003**	3.1 (1.7)	2 (0.6)	**0.002**

Significant *p* values are in bold. UWS, unresponsive wakefulness syndrome; MCS, minimally conscious state; TBI, traumatic brain injury; HIBI, hypoxic-ischemic brain injury; CRS-R, Coma Recovery Scale—Revised; GOSE, Glasgow Outcome Scale—Extended. Values are means (SD).

**Table 2:** NFL levels at 1–3 and 6 months post-injury.

Patients	Number of NFL Samples at 1–3 Months Post-Injury	Number of NFL Samples at 6 Months Post-Injury	NFL Level at 1–3 Months Post-Injury (pg/mL)	NFL Level at 6 Months Post-Injury (pg/mL)	*U*	*p*
All patients	70	52	1729 (982, 2679)	426 (241, 959)	586	<0.0001
UWS	45	22	2080 (1308, 3658)	434 (250, 997)	130	<0.0001
MCS	25	30 *	1325 (934, 3269)	426 (238, 919)	152	<0.0001
TBI	48	35	1383 (954, 2165)	337 (212, 557)	280	<0.0001
HIBI	22	17	2545 (1708, 3574)	818 (421, 1031)	48	<0.0001

NFL levels are expressed as median (25th, 75th percentiles). * Includes patients with MCS and emerged from MCS. NFL, neurofilament light chain; UWS, unresponsive wakefulness syndrome; MCS, minimally conscious state; TBI, traumatic brain injury; HIBI, hypoxic-ischemic brain injury.

## Data Availability

The datasets generated during the current study are available from the corresponding author on reasonable request.

## Supplementary Materials

The following are available online at https://www.mdpi.com/article/10.3390/brainsci11081068/s1, Table S1: Patients’ demographic and clinical details.
