# Supplementary material for: Sustained Axonal Degeneration in Prolonged Disorders of Consciousness
Source: Brain Sci. 2021 Aug 14;11(8):1068. doi: 10.3390/brainsci11081068 (PMC8394581; doi:10.3390/brainsci11081068)
Supplement: Supplementary file 1 [file brainsci-11-01068-s001.zip › brainsci-1302272-supplementary.pdf]

Supplementary Table S1. Patients' demographic and clinical details.

Pt	Sex	Age (y)	Brain injury	Time since brain injury (days)	DOC	CRS-R score	Main CT and / or MRI findings*
1	M	37	HIBI	65	UWS	3	Bi-hemispheric hypodensity
2	M	65.4	HIBI	46	UWS	2	None
3	M	56.4	TBI	46	MCS	7	Left hemispheric subdural hematoma
4	M	19	TBI	30	UWS	5	Diffuse axonal injury; cerebral edema
5	M	42.8	TBI	33	MCS	18	Diffuse axonal injury
6	M	24.3	HIBI	64	UWS	6	None
7	F	34.8	HIBI	37	UWS	4	None
8	M	29.5	TBI	30	MCS	10	Left hemispheric epidural hematoma
9	F	18.8	TBI	57	MCS	18	Left frontal epidural and subdural hematoma; cerebral edema
10	M	26	TBI	43	MCS	15	Right fronto-temporal cortical contusions
11	M	20.4	TBI	90	UWS	4	Right frontal epidural hematoma; multiple cortical contusions; cerebral edema
12	F	30.4	TBI	47	UWS	6	Diffuse axonal injury
13	M	29.8	TBI	49	MCS	11	Bilateral frontal cortical contusions; cerebral edema
14	M	39.7	TBI	38	MCS	8	Right temporo-parietal epidural hematoma
15	M	22.9	TBI	30	MCS	8	Multiple cortical contusions; intraventricular hemorrhage
16	M	22.5	TBI	16	UWS	3	Left hemispheric epidural hematoma; ischemic lesions in the brainstem
17	M	46.1	TBI	58	UWS	2	Bi-hemispheric epidural hematoma; diffuse axonal injury
18	M	27.7	TBI	33	MCS	14	Diffuse axonal injury
19	M	17.8	TBI	33	MCS	12	Left lenticular nucleus hemorrhage
20	M	22.1	TBI	35	MCS	14	Right hemispheric epidural and subdural hematoma
21	M	51.1	TBI	42	UWS	2	Diffuse axonal injury
22	M	43.7	HIBI	30	UWS	5	Bilateral hypoxic lesions in the occipital cortex, thalamus and hippocampus
23	M	18.7	TBI	40	UWS	5	Multiple cortical contusions; cerebral edema
24	M	34.1	HIBI	65	UWS	2	Cerebral edema
25	M	18.2	TBI	52	MCS	18	Bilateral frontal subdural hematoma
26	M	60.9	TBI	24	UWS	2	Right hemispheric epidural hematoma
27	M	24.2	HIBI	40	UWS	3	Bi-hemispheric hypodensity; cerebral edema
28	M	58.3	TBI	21	UWS	6	Left hemispheric epidural hematoma; right hemispheric subdural hematoma
29	M	49	TBI	28	UWS	6	Bilateral frontal intraparenchymal hemorrhage
30	M	42.4	TBI	32	UWS	4	Left lenticular nucleus hemorrhage; multiple cortical contusions
31	M	18.1	TBI	42	MCS	6	Diffuse axonal injury
32	M	48.2	HIBI	29	UWS	5	Cerebral edema
33	M	58.6	TBI	70	UWS	4	Right hemispheric subdural hematoma
34	M	33.3	TBI	25	UWS	2	Diffuse axonal injury
35	M	24.2	HIBI	39	UWS	6	Cerebral edema
36	M	19.1	HIBI	50	UWS	4	Cerebral edema
37	M	63.4	HIBI	19	UWS	4	None
38	M	38.4	TBI	90	UWS	7	Multiple cerebral and cerebellar contusions

39	F	40.1	TBI	38	UWS	3	Intraventricular hemorrhage
40	M	48.6	HIBI	87	UWS	8	Cerebral edema
41	M	51.4	HIBI	90	MCS	10	Cerebral edema
42	M	52.1	TBI	79	MCS	12	Bilateral hemispheric subarachnoid hemorrhage; right hemispheric epidural hematoma
43	F	64.6	TBI	71	MCS	13	Left hemispheric subdural hematoma
44	F	55.4	TBI	50	MCS	12	Bilateral hemispheric subarachnoid hemorrhage; cerebral edema
45	M	20.6	TBI	59	UWS	7	Right hemispheric subdural hematoma
46	F	64	HIBI	24	UWS	5	None
47	M	56.8	HIBI	84	UWS	6	Hypoxic white matter lesions
48	M	64	TBI	74	UWS	3	Bilateral hemispheric subarachnoid hemorrhage
49	F	45.5	HIBI	45	UWS	4	None
50	M	53.6	TBI	43	UWS	2	Bilateral hemispheric subarachnoid hemorrhage
51	M	45.0	TBI	33	UWS	2	Right hemispheric cortical contusions
52	M	53.8	HIBI	58	UWS	4	Cerebral edema
53	M	22.2	TBI	73	MCS	14	Right frontal intraparenchymal hemorrhage
54	M	49	TBI	37	UWS	6	Bilateral cortical contusions
55	F	63.8	HIBI	23	UWS	5	None
56	M	38.3	HIBI	13	MCS	5	None
57	M	37.9	TBI	36	UWS	4	Diffuse axonal injury
58	M	42.9	TBI	54	UWS	3	Brainstem and thalamic hemorrhage; intraventricular hemorrhage
59	M	36.4	TBI	89	MCS	12	Left hemispheric epidural hematoma; subarachnoid hemorrhage
60	M	32.1	HIBI	31	UWS	5	Hypoxic white matter lesions
61	M	28.9	TBI	29	UWS	2	Left hemispheric subdural hematoma in the
62	M	34.1	TBI	72	UWS	6	Diffuse axonal injury; bilateral cortical contusions
63	M	28	TBI	55	MCS	13	Cerebral edema
64	F	40.4	TBI	65	MCS	10	Diffuse axonal injury
65	M	27.7	TBI	69	MCS	12	Diffuse axonal injury
66	M	18.7	TBI	30	UWS	5	Diffuse axonal injury
67	F	30.1	TBI	30	MCS	18	Diffuse axonal injury; right hemispheric cortical contusions
68	M	52.3	HIBI	49	UWS	6	Bilateral cortical laminar necrosis
69	F	43.6	HIBI	40	UWS	4	None
70	M	65	TBI	62	MCS	19	Right frontal intraparenchymal hemorrhage

* When available, the data are from immediately after brain injury; otherwise, the first data available is shown.

CRS-R, Coma Recovery Scale–Revised; CT, computed tomography; DOC, disorder of consciousness; F, female; HIBI, hypoxic-ischemic brain injury; M, male; MCS, minimally conscious state; MRI, magnetic resonance imaging; Pt, patient; TBI, traumatic brain injury; UWS, unresponsive wakefulness syndrome.
